# Long-Lasting Enhanced Cytokine Responses Following SARS-CoV-2 BNT162b2 mRNA Vaccination

**DOI:** 10.3390/vaccines12070736

**Published:** 2024-07-03

**Authors:** Georgiana Cabău, Medeea Badii, Andreea M. Mirea, Orsolya I. Gaal, Liesbeth van Emst, Radu A. Popp, Tania O. Crișan, Leo A. B. Joosten

**Affiliations:** 1 Department of Medical Genetics, “Iuliu Haţieganu” University of Medicine and Pharmacy, 400012 Cluj-Napoca, Romaniamirea.andreea.manuela@elearn.umfcluj.ro (A.M.M.);; 2 Department of Internal Medicine, Radboud UMC, 6525 GA Nijmegen, The Netherlands

**Keywords:** mRNA vaccine, cytokine responses, proteomics, innate immune memory, inflammatory responses

## Abstract

The mRNA vaccine against COVID-19 protects against severe disease by the induction of robust humoral and cellular responses. Recent studies have shown the capacity of some vaccines to induce enduring non-specific innate immune responses by the induction of trained immunity, augmenting protection against unrelated pathogens. This study aimed to assess whether the mRNA vaccine BNT162b2 can induce lasting non-specific immune responses in myeloid cells following a three-dose vaccination scheme. In a sample size consisting of 20 healthy individuals from Romania, we assessed inflammatory proteins using the Olink^®^ Target 96 Inflammation panel, as well as ex vivo cytokine responses following stimulations with unrelated PRR ligands. We assessed the vaccine-induced non-specific systemic inflammation and functional adaptations of myeloid cells. Our results revealed the induction of a stimulus- and cytokine-dependent innate immune memory phenotype that became apparent after the booster dose and was maintained eight months later in the absence of systemic inflammation.

## 1. Introduction

The global effort to combat the COVID-19 pandemic caused by the severe acute respiratory syndrome coronavirus 2 (SARS-CoV-2) has prompted the rapid development and widespread deployment of vaccines, including the emergency use of messenger RNA (mRNA)-based vaccines. The BNT162b2 vaccine, which uses an mRNA template for the translation of the viral spike glycoprotein of SARS-CoV-2 encapsulated in a lipid nanoparticle (LNP), has proven to be efficacious in preventing severe disease, by the induction of humoral and cellular immune responses against the spike protein [1,2,3,4,5,6]. Data from the clinical trials and epidemiological studies following vaccination have reported on systemic inflammatory adverse effects [7,8,9,10,11,12] and on the inflammatory nature of the LNP used for delivery [13], potentially contributing to adverse health outcomes. Nonetheless, additional epidemiological data on the COVID-19 vaccination have suggested that the vaccine confers non-specific protection, as evidenced by the reduced all-cause mortality [14,15]. A growing body of epidemiological and immunological research has revealed the protective effects of vaccination beyond their target infection, with heterologous, non-specific protective effects of some live attenuated vaccines persisting for up to 5 years, such as in the case of the Bacillus Calmette–Guérin vaccine [16]. This could at least be partly explained by trained immunity (TRIM) or innate immune memory, in which the long-term adaptations of the innate immune cells following an initial stimulation alters the capacity of the cells through metabolic and epigenetic reprogramming, allowing for a more robust response to future encounters with the same or different stimulus [16]. More recently, the adenoviral COVID-19 vaccination (ChAdOx1) was shown to alter cytokine and chemokine production, as well as glycolysis, a hallmark of trained immunity, suggesting the induction of TRIM [17]. Using various assessment timepoints following a two-dose regimen, studies investigating immune responses to the mRNA BNT162b2 vaccine demonstrated enhanced adaptive and innate immune responses in mice [18] and, through a systems vaccinology method, in human volunteers [19]. Some have also reported non-specific cytokine alterations in response to unrelated pathogens [20]. However, there is currently a lack of data on the extent of immune dysregulation. The present study aims to address that gap in knowledge by uniquely examining the longevity of non-specific immune responses and cytokine responses to unrelated pathogens, both following vaccination with BNT162b2 and eight months after the last dose. An understanding of the long-lasting innate immune memory elicited by mRNA vaccines has the potential to be exploited in vaccine design. This can enhance efficacy and protection against unrelated pathogens in vulnerable populations, guide future vaccination strategies, inform public health policies, and deepen our understanding of immune responses against emerging pathogens [21].

In the present study, we explored long-term non-specific immune responses following mRNA vaccination with BNT162b2. Our goal was to address two key scientific questions as follows: First, whether the vaccine induces long-term systemic inflammation, for which we performed a targeted plasma inflammatory proteomic profiling. Second, we sought to determine if the BNT162b2 vaccination alters the innate immune response towards unrelated pathogens in experimental in vitro assays as assessed by induced monocytic cytokine production following the stimulation of PBMCs with a broad range of unrelated bacterial and fungal pathogens. These experiments revealed that the BNT162b2 vaccination is not associated with systemic inflammation at any of the observed timepoints. Moreover, our data revealed that the BNT162b2 vaccine is associated with persistent altered cytokine production of myeloid cells in response to unrelated stimuli. This elevated cytokine production capacity is not transient and is observed even at eight months after the booster dose. This emphasizes the vaccine’s lasting impact and its potential for long-term non-specific protection against a broad range of pathogens, contributing to the understanding of the innate immune responses associated with mRNA vaccination. Future studies are needed to establish the generalizability of these findings, as the present study is comprised of mostly young females from Romania, and to further elucidate which component of the mRNA vaccine is accountable for the observed effects.

## 2. Materials and Methods

### 2.1. Study Design and Ethics

A total of 20 healthy healthcare workers from Romania were included in the study conducted between 2021 and 2022. All participants were White and from Romania. Three individuals had declared their COVID-19 infection at least three months prior to inclusion. These were included in the study as the standard immunization protocol was recommended regardless of previous infection status. One individual contracted COVID-19 during the study and was excluded from further analysis. No other participant had a known infection prior to or during the study. Another participant was excluded due to receiving other vaccinations during the study, while one participant refused the booster vaccine and was further excluded, and four participants were lost to follow-up. For detailed participant information, please see Supplementary Figure S1. All the participants were vaccinated with the monovalent BNT162b2 vaccine developed by Pfizer–BioNTech targeting the spike protein of the wild-type strain of SARS-CoV-2 identified in Wuhan, China, and followed the standard vaccination regimen used in Romania. Participants received the first dose in January 2021, followed by the second dose after 3 weeks and the booster shot 8 months after the second dose. No participant reported major adverse events. Blood was collected at baseline, two days prior to the first dose and, on average, seven days (5–11 days) after each vaccination, followed by eight months after the booster shot for the long-term assessment mark.

### 2.2. Plasma Separation

Whole blood was collected in EDTA-treated tubes and centrifuged at 1700 rpm for 10 min. Following centrifugation, the plasma was collected, aliquoted, and stored at −80 °C until the antibody and proteomic assays were performed.

### 2.3. Antibody Measurement

For antibody measurement, the IgG anti-S protein antibody titers were measured in all plasma samples available at baseline (*n* = 19), after dose 1 (*n* = 11), after dose 2 (*n* = 19), after the booster shot (*n* = 15), and 8 months later (*n* = 13) using the commercial ELISA kit, Human SARS-CoV-2 Spike (trimer) IgG ELISA Kit (Invitrogen, Waltham, MA, USA), according to manufacturer instructions.

### 2.4. Proteomic Analysis and Data Processing

The Olink^®^ Target 96 inflammation panel was used for investigating inflammatory plasma proteins (Table S1). The technology utilizes real-time PCR (qPCR) to simultaneously quantify a predefined panel of 92 proteins within a sample [22]. A multi-step quality control was performed to correct for intra-assay and inter-assay technical variability. The resulting Ct-values were normalized, generating NPX (normalized protein expression) values. After exclusion of the samples that did not meet QC standards (*n* = 1), encountered technical issues (*n* = 8), or were excluded during the study (see Figure S1), the remaining available samples at each timepoint were analyzed. Proteins with NPX values below the limit of detection in 30% or more of the samples (*n* = 18: GDNF, IL2, IL-20RA, IL-2RB, IL-1 alpha, IL-20, TSLP, IL-22 RA1, Beta-NGF, IL33, IL-13, ARTN, IL6, IL-24, IL-4, LIF, NRTN, IL5) were excluded and the subsequent analysis focused on the set of 74 remaining proteins.

### 2.5. Primary Cell Culture and Cytokine Measurement

Peripheral blood mononuclear cells (PBMCs) were isolated from blood using Ficoll-Paque PLUS (GE Healthcare, Chicago, IL, USA). Freshly isolated PBMCs were seeded in 96 well round-bottom microplates (Greiner Bio-One, Kremsmünster, Austria) at a concentration of 5 × 10^5^ cells/mL in the culture medium, RPMI 1640 (Sigma-Aldrich, St. Louis, MO, USA) supplemented with 1 mM pyruvate, 50 µg/mL gentamicin, and 2 mM GlutaMAX (Gibco, Waltham, MA, USA). Stimuli stocks were prepared in advance, aliquoted, and kept at −20 °C until the experiments were performed. The cells were stimulated with either LPS (1 ng/mL LPS from *E. coli* 055:B5, Sigma-Aldrich, USA), HK *Candida albicans* (1 × 10^6^ CFU/mL), HK *Staphylococcus aureus* (1 × 10^6^ CFU/mL), HK *Borrelia burgdorferi* (1 × 10^6^ CFU/mL), HK H37Rv strain *Mycobacterium tuberculosis* lysate (5 µg/mL), phytohemagglutinin (10 µg/mL), or medium control. The plates were incubated at 37 °C and 5% CO_2_ for 24 h. Following incubation, the plates were centrifuged, and the supernatants were removed and stored at −20 °C until cytokine measurement was performed. Three independent experiments were conducted for the pre-vaccination baseline, followed by three experiments for samples taken after the first dose, five experiments for samples after the second dose, four experiments after the booster dose, and two experiments at the eight-month mark. IL-1β (Interleukin 1 beta), IL-1Ra (Interleukin-1 receptor antagonist protein), IL-6 (Interleukin 6), and TNF-α (Tumor necrosis factor alpha) production were measured using commercial DuoSet ELISA kits (R&D Systems, Minneapolis, MN, USA, RD-DY201 for IL-1 beta, RD-DY206 for IL-6, RD-DY210 for TNF-alpha, and RD-DY280 for IL-1Ra) in harvested supernatants. Absorbance was measured on a BioTek Synergy HTX reader. Cytokine production was assessed concurrently across all samples at baseline, after the first dose, after the second dose, and after the booster using plate controls, while the cytokine concentrations at the eight-month mark were quantified using plate controls and inter-assay controls from previous time points.

### 2.6. Statistical Analysis

Differences in antibody levels were assessed using the Wilcoxon matched-pairs signed-rank test by comparing all the available samples at that timepoint, i.e., dose 1 (*n* = 11), dose 2 (*n* = 17), booster (*n* = 15), and 8 months (*n* = 13) to their respective baseline levels. Differences in plasma protein levels after dose 1 (*n* = 11), after dose 2 (*n* = 19), after the booster (*n* = 15), and at eight months following the booster dose (*n* = 13) were compared to their respective baseline levels using paired multiple *t*-tests with the Benjamini–Hochberg method for controlling the false-discovery rate (FDR, Q = 5%). Values with q < 0.05 were considered significant. Differences in cytokine production between each timepoint compared to baseline and between the eight-month mark and the booster timepoint were evaluated using the paired non-parametric test of Friedman with Dunn’s correction for multiple comparisons, after testing for normality of distribution using the Shapiro–Wilk and Kolmogorov–Smirnov tests. Two-tailed *p* values < 0.05 are reported.

## 3. Results

### 3.1. Study Design and Participants’ Characteristics

**Figure 1:**
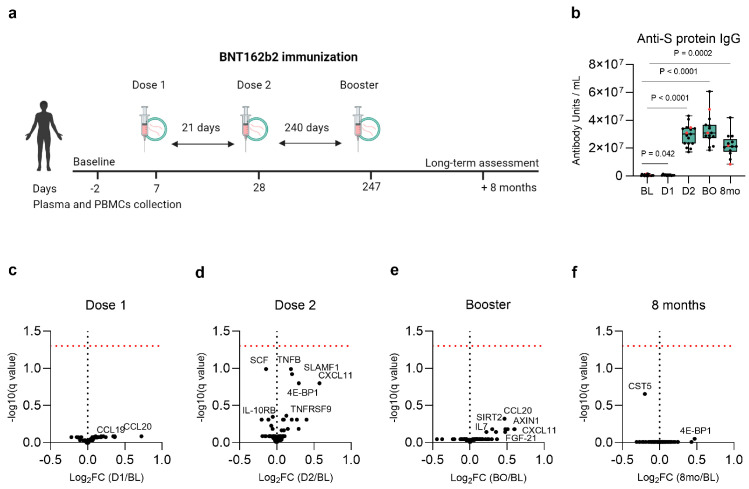
Study design and plasma inflammatory proteomic profiles following BNT162b2 vaccination. (**a**) Vaccination scheme and timing of plasma and PBMC isolation. (**b**) The anti-S IgG antibody titers at each timepoint. Individuals who declared a COVID-19 infection prior to inclusion are labeled in red. Vaccination timepoints (D1, D2, BO) and the 8-month mark values were compared to the baseline using the Wilcoxon matched-pairs signed-rank test. Boxplots with a line at the median and the 75th and 25th percentiles; whiskers showing the range of values. (**c**–**f**) Volcano plot showing the differences in the proteomic profiles following (**c**) dose 1 (*n* = 11), (**d**) dose 2 (*n* = 19), (**e**) booster vaccination (*n* = 15), and (**f**) 8 months later (*n* = 13) compared to baseline. The red dashed line represents the threshold of significance (−log10(0.05)). Nominally significant proteins were labeled. Comparisons were made using the paired multiple *t*-tests with the FDR (Q = 5%) method of Benjamini and Hochberg for multiple comparisons. BL: baseline, D1: dose 1, D2: dose 2, BO: booster, 8mo: eight months.

Participants received a three-dose vaccination regimen with three weeks (21 days) between dose 1 and dose 2, and then eight months (240 days) between dose 2 and the booster shot. Plasma and PBMCs stimulation experiments were performed at five different timepoints as follows: two days before vaccination (mean 2.1, 2.7 SD), seven days (mean 7.4, 0.9 SD) after the first dose, after the second dose (day 28 after the first vaccination), and after the booster dose (day 247 after the first vaccination), as well as 8 months (mean 257.5 days, 15.1 SD) following completion of the BNT162b2 vaccination scheme with the last dose. At the time of inclusion, the participants were between the ages of 26 and 49 (mean age 29.7, 5.4 SD), with 18 women and 2 men. No participant declared having an acute or chronic disease. The study design is shown in Figure 1a, while the detailed participant information is in Figure S1.

### 3.2. Baseline, Post-Vaccination, and Long-Term Anti-S Protein IgG Antibody Concentration

To confirm that the humoral response known to be induced by vaccination was present, we measured plasma anti-S protein IgG antibody titers at baseline before vaccination and after the standard regimen of immunization with the three doses of BNT162b2. Using a paired statistical analysis for all the available samples at a specific timepoint, we compared antibody titers to baseline levels. A moderate increase in the anti-S protein IgG antibody titers following the first dose compared to baseline values was observed (Figure 1b). Subsequent vaccination strongly increased the antibody levels, confirming the anticipated adaptive immune response. Long-term assessment at the eight-month mark compared to baseline values revealed maintenance of antibody production after completion of the vaccination scheme, although this timepoint tended to show lower antibody values compared to those following the second and the booster dose (Figure 1b).

### 3.3. No Lasting Changes in the Plasma Inflammatory Proteome Following Vaccination

Next, we focused on non-specific immune responses potentially induced by vaccination, examining the plasma concentration of 74 inflammatory markers seven days after each vaccination and eight months later after the final dose. Several of the following cytokines, chemokines, and immune regulatory proteins tended to be differentially expressed: CCL19 and CCL20 chemokines after the first dose (Figure 1c), TNFB, SLAMF1, CXCL11, 4E-BP1, TNFRSF9, SCF, and IL-10RB following the second dose (Figure 1d), CCL20, CXCL11, AXIN1, FGF-21, SIRT2, and IL7 after the booster dose (Figure 1e), and CST-5 and 4E-BP1 at the eight-month mark, compared to baseline levels (Figure 1f). However, none of the proteins remained significant following multiple-testing correction at any of the observed time points after immunization compared to prior baseline values. Arunachalam et al. [19] used the same proteomic panel to assess plasma proteins following the first two doses of BNT162b2 and found that the CXCL10 and IFN-γ concentrations increased on day 1 and day 2 after the first vaccine dose, with a stronger response observed for IFN-γ after the second dose, as well as increased MCP-2, CXCL9, CXCL10, and CXCL11 within the first two days after the second vaccination [19]. However, it is important to note that these proteins returned to baseline values by day 7 [19], the timepoint investigated in our study. Similarly, the same transient response was found in the mice sera, elevated cytokine and chemokine levels were detected 6 h post-immunization, returning to baseline levels by day 3 [18]. Consistent with these findings [18,19], our comparison of the plasma inflammatory profile after the second immunization to the first dose showed a similar trend. Among the nominally significant proteins, the chemokines MCP-1, CXCL9, CXCL10, and CXCL11 tended to increase, suggesting normalization by day 7 (Figure S3a). Other proteins tended to be expressed when comparing the booster dose to the second dose or the eight-month mark to the booster dose, but none remained significant after multiple-testing correction (Figure S3b,c).

### 3.4. BNT162b2 Vaccination Is Associated with Persistent Cytokine Alterations in Stimulated PBMCs

Next, we measured ex vivo monocytic cytokine production in the culture supernatants after 24 h stimulation with a broad range of toll-like receptors (TLR) engaging pathogen-associated molecular patterns *S. aureus* (TLR2) [23], *M. tuberculosis* (TLR2/TLR6) [24], *B. burgdorferi* (TLR2/TLR1) [25], *C. albicans* (TLR2/TLR4) [26], LPS, and PHA lectin signaling through the activation of TLR4 [27,28].

The assessment was performed an average of one week after each vaccination and 8 months post-completion of the immunization regimen. Comparisons of each timepoint were drawn to the baseline pre-vaccination responses, and the final eight-month timepoint was also compared to the post-booster responses.

**Figure 2:**
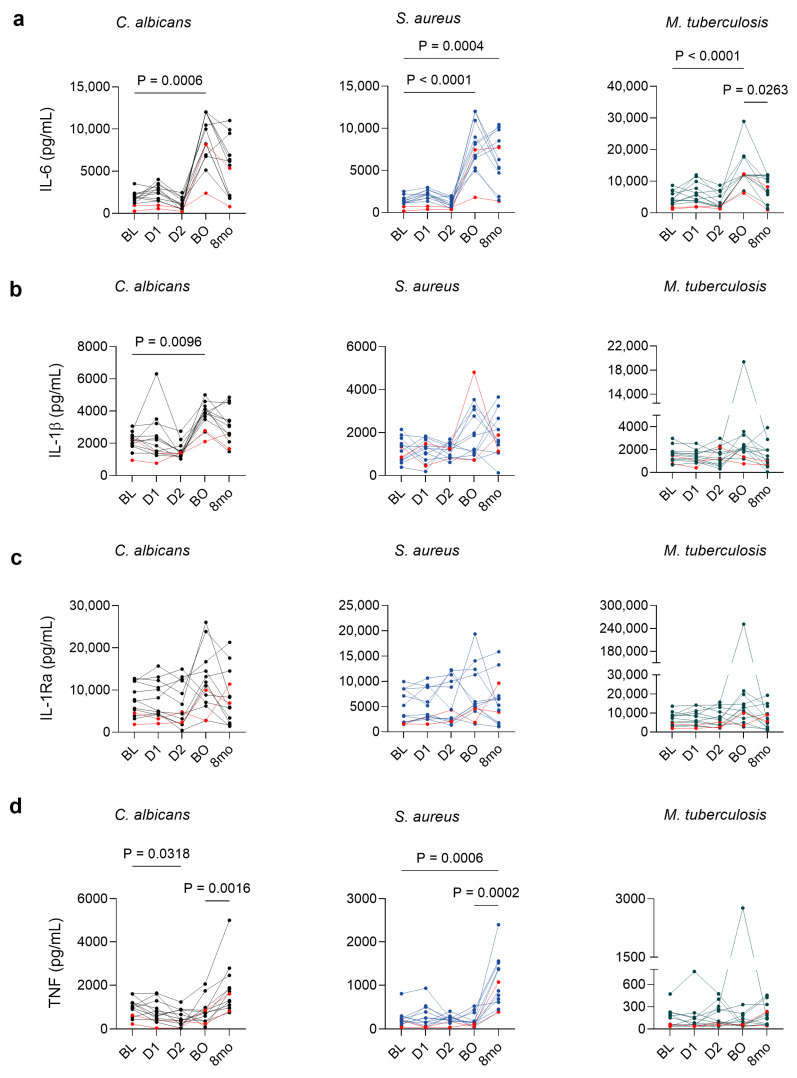
Enhanced cytokine production following BNT162b2 vaccination in response to unrelated pathogens. Production of (**a**) IL-6, (**b**) *IL*-1β, (**c**) IL-1Ra, and (**d**) TNF-α in PBMCs from healthy volunteers (*n* = 13) in response to *C. albicans*, *S. aureus*, and *M. tuberculosis* stimulation at baseline, following dose 1, dose 2, the booster dose and 8 months later. Each dot with a line represents an individual sample. Individuals who declared COVID-19 infection prior to inclusion are labeled in red. Paired data were analyzed by comparing each timepoint to the baseline and booster to the eight-month mark. Friedman’s test with Dunn’s correction for multiple comparisons was used. Two-tailed *p* values < 0.05 are shown. BL: baseline, D1: dose 1, D2: dose 2, 8mo: eight months.

For *C. albicans*, *S. aureus*, *M. tuberculosis*, LPS, *B. burgdorferi*, and PHA, IL-6 showed significantly elevated concentrations after booster administration compared to baseline, which were maintained heightened at the eight-month mark, except for the *M. tuberculosis* for which it had started to wane (Figure 2a and Figure S2a). IL-1β showed a significant induction for *C. albicans* following the booster dose (Figure 2b) and for PHA as well after the booster dose, which was also maintained eight months later (Figure S2b). Significant variations in the anti-inflammatory IL-1Ra production were evident only upon LPS stimulation, where it showed a decrease at eight months compared to the post-booster concentrations (Figure 2c and Figure S2c). TNF-α production was elevated at the eight-month mark for *C. albicans* and *S. aureus*, compared to both baseline and post-booster concentrations (Figure 1d and Figure S2d). No difference in cytokine production was observed for the unstimulated cells (Figure S2).

## 4. Discussion

In the present study, we assessed whether the BNT162b2 vaccine induces lasting non-specific immune responses that can potentially impact the safety and development of mRNA-based therapeutics. This study uniquely addresses the longevity of non-specific systemic inflammation and altered cytokine responses. We found no evidence for the persistence of systemic inflammation, previously reported to be potentially induced immediately within the first few days [7,8,9,10,11,12]. We assessed 74 non-specific plasma circulating inflammatory markers whose concentrations were determined, on average, seven days following each vaccination, and we found no significant differences compared to the pre-vaccination values. A change in the plasma inflammatory proteome was also not apparent at the later eight-month timepoint. Our findings align with the previous studies examining the inflammatory markers seven to fourteen days after BNT162b2 vaccination, which found no significant evidence for increased inflammatory markers associated with vaccination at the observed time intervals [20]. Other studies showed normalization of the inflammatory markers within the first three days [18] and seven days [19], respectively. Additionally, one report observed an immediate and transient increase in serum chemokines and cytokines related to the innate and adaptive responses within 24 h following the vaccination [29]. While the inflammatory signature tended to return to baseline levels, a few inflammatory markers associated with the myeloid compartment remained elevated one month after the booster dose [29]. Among these markers, CCL3, CCL4, and IL-8 were included in our proteomic panel but were not differentially expressed after the booster dose or eight months later. These discrepancies could be due to differences in timepoints of assessment, method of detection, and statistical analyses between the studies, as well as pre-analytical variation due to different biological sample types. For instance, serum samples generally show higher levels of these chemokines compared to EDTA plasma samples due to their release from platelets during the clotting process [30]. While transient systemic inflammation in the initial days post-immunization cannot be ruled out, collectively, our data support the overall safety profile of the BNT162b2 vaccine, demonstrating no long-term inflammatory side effects as measured by the plasma circulating inflammatory markers.

The long-term adaptation of the innate immune system marked by an enhanced cytokine response to subsequent homologous or heterologous challenges, termed trained immunity, has been shown to mediate the protective effects of some vaccines towards unrelated pathogens [31,32]. While reversible, the induction of innate immune memory involves the alterations of the metabolic and epigenetic landscape of myeloid cells, facilitating delayed and finely tuned immune responses [33].

In this study, PBMCs from vaccinated individuals displayed an enhanced cytokine response following ex vivo stimulation with bacterial and fungal ligands, which cannot be explained by heterologous immunity and epitope cross-reactivity. The signaling cascade triggered by the engagement of TLR2 and TLR4 receptors led to the recruitment of adaptor proteins, such as MyD88, ultimately activating the NF-κB and AP-1 transcription factors which drive the transcription of inflammatory mediators [34]. The sustained enhancement of IL-6, IL-1β, and TNF-α production in response to TLR2 and TLR4 agonists observed in our study suggests that broad alterations of these immune pathways could play a role in the innate immune response of the BNT162b2 vaccine. Investigations of the induction of trained immunity by the BNT162b2 vaccine have examined differential cytokine production and chromatin accessibility after the second dose. Some reported no apparent changes [35], while others demonstrated that consecutive vaccinations primed immune responses by altering the chromatin landscape and boosted cytokine production in response to TLR7/8 ligands, although these effects were transient [12] and could be due to an acute immune response to vaccination. These data align with our findings that exhibited no significant differential cytokine response one week after the first two doses of vaccination.

Of high interest, our data revealed that the altered cytokine response became apparent after the booster dose and tended to be either maintained or even heightened at the eight-month mark for all stimulations, except for *M. tuberculosis* for which it started to wane. This information is relevant as it suggests this enhancement of innate responses is not short-lived but maintained for at least 8 months, which was a previously unassessed timepoint in relation to PBMC cytokine production capacity post-vaccination. Another report indicated a slight increase in cytokine production in response to unrelated pathogens long after the second vaccination and before the booster shot with no significant change at four weeks after the booster vaccination [20]. Due to the absence of data before the booster vaccination in our study, it remains indeterminate whether the altered phenotype observed here was induced by the booster vaccination itself and maintained long-term, or whether it developed as a delayed response already after the second dose [20].

It is crucial to note that both the BioNTech and Moderna COVID-19 vaccines use the same N1-methyl-pseudouridine-modified RNA (modRNA) with low reactogenicity and immunogenicity, and they are formulated in LNPs that contain different ionizable lipids but having similar chemical structures (ALC-0315 for BioNTech and SM-102 in Moderna) [36]. While the LNPs demonstrated robust intrinsic adjuvant capacity and induction of IL-1 cytokines [37], treatment with modRNA formulated in SM-102 containing LNP as well as the empty vector can generate both the signals required for NLRP3 inflammasome activation, leading to a potent IL-1β release in human PBMCs [37]. The recombinant S-protein was shown to prime human monocytes for the induction of IL-1β [38], which could be hypothesized to play a role in the enhanced cytokine production following stimulation observed in our study. However, this does not fully recapitulate the in vivo setting in which the mRNA is translated to produce the S-protein. Whether the altered cytokine response observed in our study is due to the mRNA component, the S-protein or the LNP particle remains to be determined by studies investigating the training potential of the empty vector, as well as modRNA-derived S-protein, providing crucial knowledge for future mRNA therapeutics and vaccine design.

Our study is subject to several limitations that should be considered. The cohort is relatively small and predominately comprises females (90%), therefore sex-specific effects cannot be excluded. However, when we conducted an analysis focused exclusively on female participants, no differences were observed. All participants included in the study are of Eastern European descent. Given that immune responses can vary between different populations based on genetic background and different lifestyles, the conclusions of this study cannot be definitively extrapolated to different populations without further investigation. The relatively young age of the cohort could also pose a limitation, and it is conceivable that a study conducted in older individuals may provide different insights. Ruling out asymptomatic COVID-19 infections during the study was difficult and based on routine testing upon contact or clinical suspicion using rapid antigen tests and qPCR. Notably, three participants disclosed a history of mild or asymptomatic COVID-19 infection at least three months prior to inclusion in the study. One of them was not included in the cytokine responses dataset due to being lost to follow-up. For the remaining two participants, no disparities were detected in cytokine production or the inflammatory proteomes when compared to the rest of the participants. For transparency, we have highlighted in red the participants who had a prior COVID-19 infection. Their inclusion in the analysis and lack of data point deviation contribute to the argument of the vaccine-specific effects revealed by the study and not due to COVID-19 infection. The data presented in this paper raise the discussion of whether the long-term broad effects of BNT162b2 vaccination correspond to a trained immunity-like process, in which the long-lasting hematopoietic stem cells (HSCs) undergo epigenetic and metabolic reprogramming and give rise to monocytes exhibiting enhanced responsiveness [39]. This study paves the way for future exploration of the epigenetic and metabolic adaptations underlying enhanced cytokine production in order to comprehensively assess the role of trained immunity following BNT162b2 vaccination.

## 5. Conclusions

In conclusion, our results show that the BNT162b2 vaccine exhibits long-lasting altered cytokine responses upon stimulation, which can persist for at least 8 months. Studies investigating long-term metabolic and epigenetic alterations following vaccination could help elucidate whether a trained immunity signature underlies these changes. Our results suggest that the BNT162b2 vaccine could afford protection in infection models with these stimuli bearing significant implications for vaccine development. Persistent systemic inflammation was also not apparent at any of the timepoints following vaccination. Our findings hold particular significance given that the mRNA-based vaccine technology has reached clinical validity in the context of COVID-19, but its continuous development addresses a wide spectrum of infectious diseases including Lyme [40], malaria, tuberculosis, influenza, and numerous others [41], as well as non-infectious diseases [42].

## Data Availability

The raw data supporting the conclusions of this article will be made available by the authors on request.

## Supplementary Materials

The following supporting information can be downloaded at: https://www.mdpi.com/article/10.3390/vaccines12070736/s1, Figure S1: Age, sex and exclusion criteria of participants during the study. Figure S2: Production of (a) IL-6, (b) IL-1β, (c) IL-1Ra, and (d) TNF-α in PBMCs from healthy volunteers (*n* = 13) in response to LPS, B. burgdorferi, PHA and medium control at baseline, following dose 1, dose 2, the booster dose and 8 months later. Figure S3: Volcano plot showing the differences in the proteomic profiles between vaccinations (a) dose 2 vs. dose 1 (*n* = 11), (b) booster vs. dose 2 (*n* = 15), (c) eight-months vs. booster vaccination (*n* = 13). Table S1: Protein assay list included in Olink^®^ Target 96 Inflammation.
