# Supplementary material for: Long-Lasting Enhanced Cytokine Responses Following SARS-CoV-2 BNT162b2 mRNA Vaccination
Source: Vaccines (Basel). 2024 Jul 3;12(7):736. doi: 10.3390/vaccines12070736 (PMC11281652; doi:10.3390/vaccines12070736)
Supplement: Supplementary file 1 [file vaccines-12-00736-s001.zip › vaccines-3054424-supplementary.pdf]

Supplementary Materials

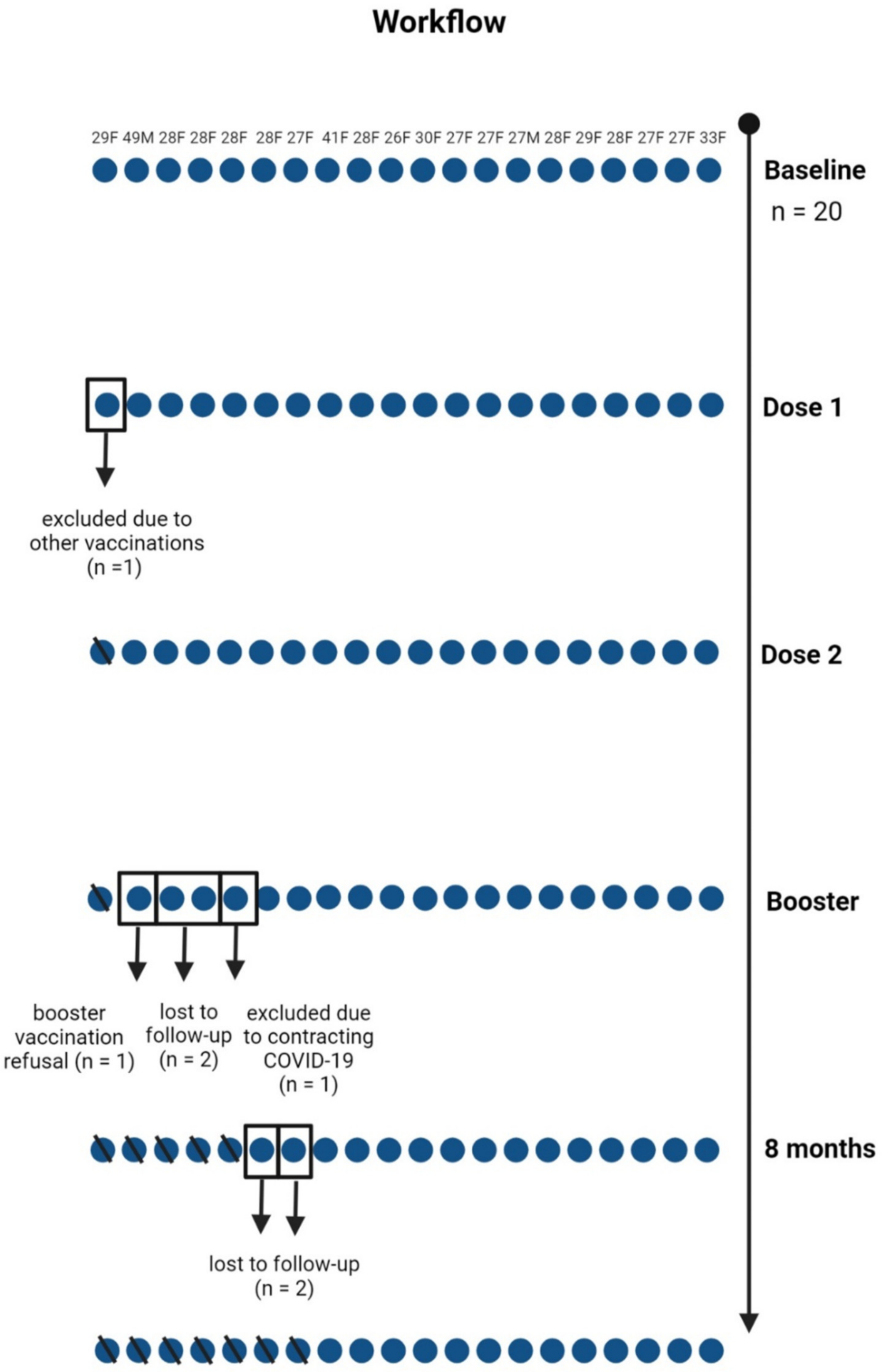

Figure S1. Age, sex and exclusion criteria of participants during the study.

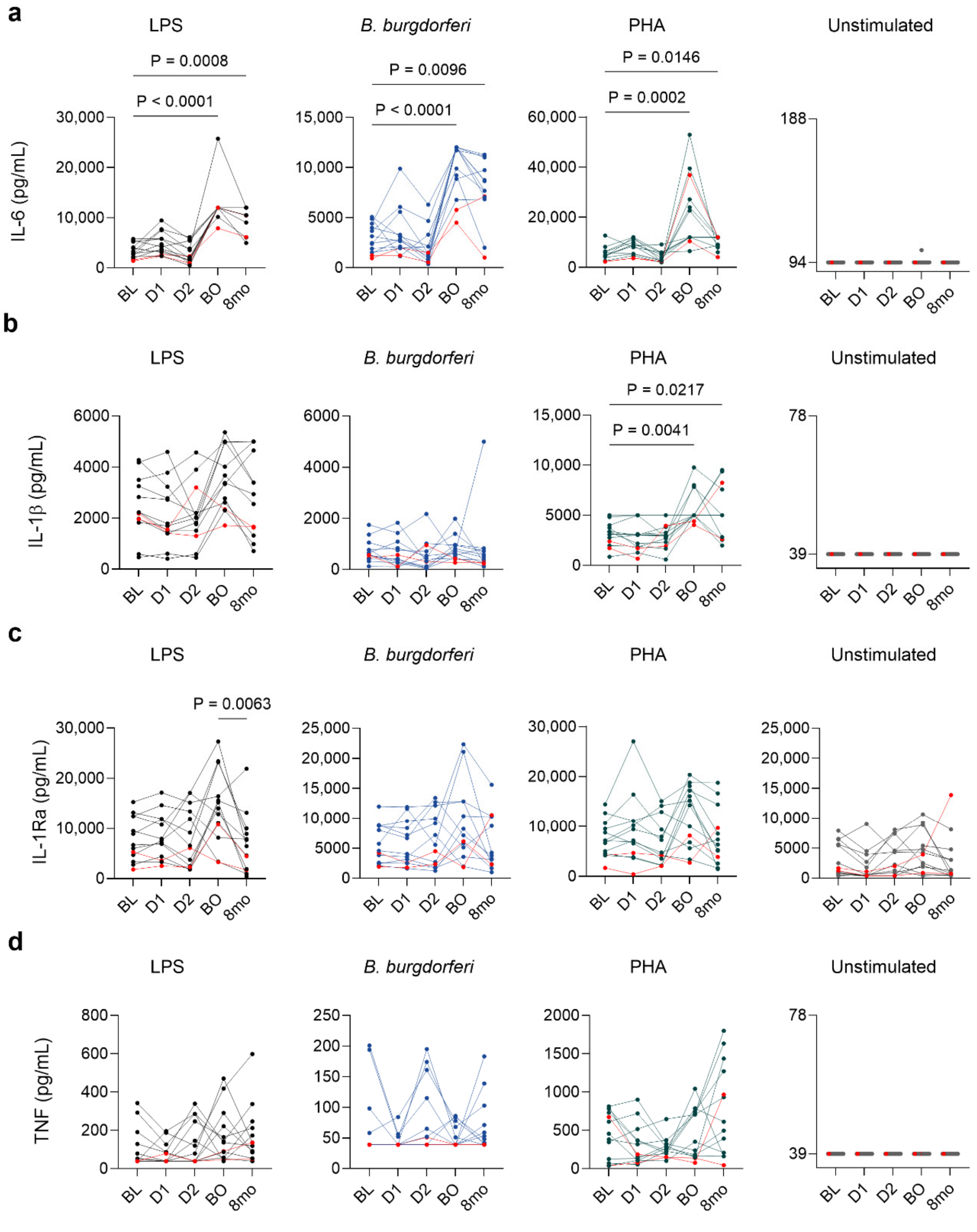

Figure S2. Production of (**a**) IL-6, (**b**) IL-1β, (**c**) IL-1Ra, and (**d**) TNF in PBMCs from healthy volunteers (n=13) in response to LPS, B. burgdorferi, PHA and medium control at baseline, following dose 1, dose 2, the booster dose and 8 months later. Each dot with line represents an individual sample. Individuals who declared COVID-19 infection prior to inclusion are labeled in red. Paired data was analysed by comparing each time point to the baseline and booster to the eight-month mark. Friedman's test with Dunn's correction for multiple comparisons was used. Two-tailed P values < 0.05 are shown. BL: baseline, D1: dose 1, D2: dose 2, 8mo: eight-months.

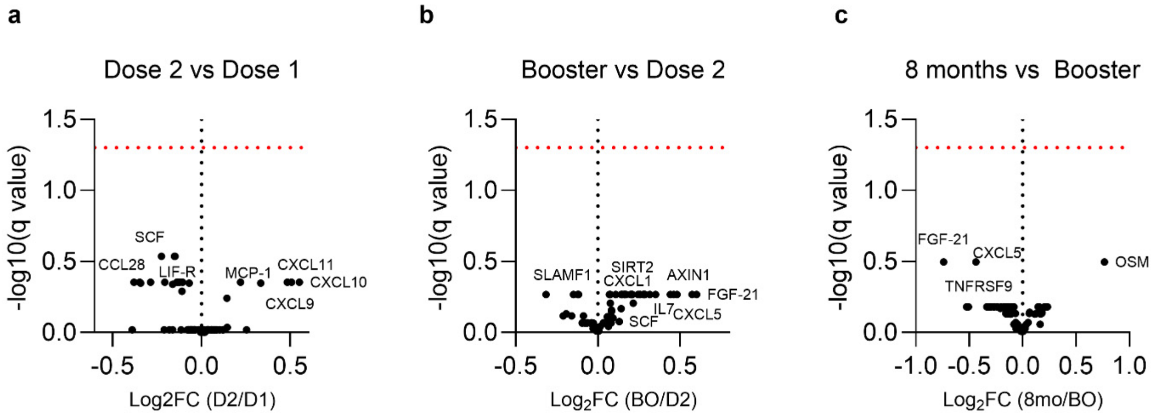

Figure S3. (**a**–**c**) Volcano plot showing the differences in the proteomic profiles between vaccinations (**a**) dose 2 vs dose 1 (n = 11), (**b**) booster vs dose 2 (n = 15), (**c**) eight-months vs booster vaccination (n = 13). The red dashed line represents the threshold of significance (-log10(0.05)). Nominally significant proteins were labeled. Comparisons were made using the paired multiple t-tests with the FDR (Q = 5%) method of Benjamini and Hochberg for multiple comparisons. BL: baseline, D1: dose 1, D2: dose 2, BO: booster, 8mo: eight-months.

Table S1. Protein assay list included in Olink Target 96 Inflammation.

Protein assay	UniProt code
Adenosine Deaminase (ADA)	P00813
Artemin (ARTN)	Q5T4W7
Axin-1 (AXIN1)	O15169
Beta-nerve growth factor (Beta-NGF)	P01138
Caspase-8 (CASP-8)	Q14790
C-C motif chemokine 3 (CCL3)	P10147
C-C motif chemokine 4 (CCL4)	P13236
C-C motif chemokine 19 (CCL19)	Q99731
C-C motif chemokine 20 (CCL20)	P78556
C-C motif chemokine 23 (CCL23)	P55773
C-C motif chemokine 25 (CCL25)	O15444
C-C motif chemokine 28 (CCL28)	Q9NRJ3
CD40L receptor (CD40)	P25942
CUB domain-containing protein 1 (CDCP1)	Q9H5V8
C-X-C motif chemokine 1 (CXCL1)	P09341
C-X-C motif chemokine 5 (CXCL5)	P42830
C-X-C motif chemokine 6 (CXCL6)	P80162
C-X-C motif chemokine 9 (CXCL9)	Q07325
C-X-C motif chemokine 10 (CXCL10)	P02778
C-X-C motif chemokine 11 (CXCL11)	O14625
Cystatin D (CST5)	P28325
Delta and Notch-like epidermal growth factor-related receptor (DNER)	Q8NFT8
Eotaxin (CCL11)	P51671
Eukaryotic translation initiation factor 4E-binding protein 1 (4E-BP1)	Q13541
Fibroblast growth factor 21 (FGF-21)	Q9NSA1
Fibroblast growth factor 23 (FGF-23)	Q9GZV9
Fibroblast growth factor 5 (FGF-5)	Q8NF90
Fibroblast growth factor 19 (FGF-19)	O95750
Fms-related tyrosine kinase 3 ligand (Flt3L)	P49771
Fractalkine (CX3CL1)	P78423
Glial cell line-derived neurotrophic factor (GDNF)	P39905
Hepatocyte growth factor (HGF)	P14210
Interferon gamma (IFN-gamma)	P01579
Interleukin-1 alpha (IL-1 alpha)	P01583
Interleukin-2 (IL-2)	P60568
Interleukin-2 receptor subunit beta (IL-2RB)	P14784
Interleukin-4 (IL-4)	P05112
Interleukin-5 (IL5)	P05113
Interleukin-6 (IL6)	P05231
Interleukin-7 (IL-7)	P13232

Interleukin-8 (IL-8)	P10145
Interleukin-10 (IL10)	P22301
Interleukin-10 receptor subunit alpha (IL-10RA)	Q13651
Interleukin-10 receptor subunit beta (IL-10RB)	Q08334
Interleukin-12 subunit beta (IL-12B)	P29460
Interleukin-13 (IL-13)	P35225
Interleukin-15 receptor subunit alpha (IL-15RA)	Q13261
Interleukin-17A (IL-17A)	Q16552
Interleukin-17C (IL-17C)	Q9P0M4
Interleukin-18 (IL-18)	Q14116
Interleukin-18 receptor 1 (IL-18R1)	Q13478
Interleukin-20 (IL-20)	Q9NYY1
Interleukin-20 receptor subunit alpha (IL-20RA)	Q9UHF4
Interleukin-22 receptor subunit alpha-1 (IL-22 RA1)	Q8N6P7
Interleukin-24 (IL-24)	Q13007
Interleukin-33 (IL-33)	O95760
Latency-associated peptide transforming growth factor beta-1 (LAP TGF-beta-1)	P01137
Leukemia inhibitory factor (LIF)	P15018
Leukemia inhibitory factor receptor (LIF-R)	P42702
Macrophage colony-stimulating factor 1 (CSF-1)	P09603
Matrix metalloproteinase-1 (MMP-1)	P03956
Matrix metalloproteinase-10 (MMP-10)	P09238
Monocyte chemotactic protein 1 (MCP-1)	P13500
Monocyte chemotactic protein 2 (MCP-2)	P80075
Monocyte chemotactic protein 3 (MCP-3)	P80098
Monocyte chemotactic protein 4 (MCP-4)	Q99616
Natural killer cell receptor 2B4 (CD244)	Q9BZW8
Neurotrophin-3 (NT-3)	P20783
Neurturin (NRTN)	Q99748
Oncostatin-M (OSM)	P13725
Osteoprotegerin (OPG)	O00300
Programmed cell death 1 ligand 1 (PD-L1)	Q9NZQ7
Protein S100-A12 (EN-RAGE)	P80511
Signaling lymphocytic activation molecule (SLAMF1)	Q13291
SIR2-like protein 2 (SIRT2)	Q8IXJ6
STAM-binding protein (STAMBP)	O95630
Stem cell factor (SCF)	P21583
Sulfotransferase 1A1 (ST1A1)	P50225
T cell surface glycoprotein CD6 isoform (CD6)	Q8WWJ7
T-cell surface glycoprotein CD5 (CD5)	P06127
T-cell surface glycoprotein CD8 alpha chain (CD8A)	P01732
Thymic stromal lymphopoietin (TSLP)	Q969D9
TNF-beta (TNFB)	P01374
TNF-related activation-induced cytokine (TRANCE)	O14788

TNF-related apoptosis-inducing ligand (TRAIL)	P50591
Transforming growth factor alpha (TGF-alpha)	P01135
Tumor necrosis factor (Ligand) superfamily, member 12 (TWEAK)	O43508
Tumor necrosis factor (TNF)	P01375
Tumor necrosis factor ligand superfamily member 14 (TNFSF14)	O43557
Tumor necrosis factor receptor superfamily member 9 (TNFRSF9)	Q07011
Urokinase-type plasminogen activator (uPA)	P00749
Vascular endothelial growth factor A (VEGF-A)	P15692
